# Core Outcome Set Development for Tension-Type Headache Treatment Using Traditional Chinese Medicine: Protocol for a Delphi Consensus Study

**DOI:** 10.2196/63481

**Published:** 2025-02-05

**Authors:** Guojing Fu, Yunmeng Chen, Xiao Liang, Chunli Guo, Xueming Fan, Xiao Gong, Wenjie Chen, Jing Teng, Jun Tang, Xing Liao, Jingjing Wei, Yunling Zhang

**Affiliations:** 1 Xiyuan Hospital China Academy of Chinese Medical Sciences Beijing China; 2 Affiliated Hospital of Shandong University of Traditional Chinese Medicine Jinan China; 3 Chongqing Traditional Chinese Medicine Hospital Chongqing China; 4 Center for Evidence-based Chinese Medicine Institute of Basic Research in Clinical Medicine China Academy of Chinese Medical Sciences Beijing China

**Keywords:** tension-type headache, core outcome set, traditional Chinese medicine, systematic review, Delphi, protocol

## Abstract

**Background:**

Tension-type headache (TTH) is the most common type of headache and the second most common health-related complaint among children and adults. Traditional Chinese medicine (TCM) offers unique therapeutic benefits in treating TTH. However, the lack of standardized evidence—such as inconsistencies in outcome selection and reporting in clinical studies, a lack of consensus on outcomes and measures, high risks of selective reporting bias, and missing data—has limited the development of robust evidence supporting the efficacy of TCM in treating TTH. Therefore, establishing a core outcome set (COS) is crucial for standardizing TCM clinical studies for TTH, thereby enhancing the quality and comparability of research findings.

**Objective:**

This study aims to develop a COS for future clinical studies on the treatment of TTH with TCM.

**Methods:**

The COS will be developed through the following 3 stages. First, systematic reviews and semistructured interviews will be conducted to identify potential essential outcomes, which will be evaluated by the steering committee to finalize a preliminary list of outcomes. Data will be processed using thematic analysis to ensure comprehensive coverage of relevant outcomes. Second, a 2-round Delphi survey will be conducted, inviting stakeholders, including health care experts and patients with tension-type headaches, to determine the importance of each outcome. Statistical analysis will be used to assess the level of consensus and prioritize outcomes based on predefined criteria. Third, a face-to-face consensus meeting will be held to finalize the COS and recommend measurement times for each outcome. Key outcomes will be interpreted based on their clinical relevance and feasibility of measurement, ensuring the COS is comprehensive and applicable in clinical settings.

**Results:**

The protocol has been registered in PROSPERO, with the review commencing on October 1, 2024, and anticipated results by November 15, 2024. The systematic reviews will be finalized, followed by the Delphi survey and consensus conference in late 2024 and early 2025. The COS findings will be reported per COS-STAR (Core Outcome Set–STAndards for Reporting) guidelines, published in an international journal, presented at conferences, and disseminated to participants for clinical application.

**Conclusions:**

This study is necessary as developing a COS for future TCM clinical studies in the treatment of TTH can maximize the value of data from individual trials and provide high-quality research evidence.

**Trial Registration:**

Core Outcome Measures in Effectiveness Trials Initiative 1473; https://tinyurl.com/3ts62s2p

**International Registered Report Identifier (IRRID):**

PRR1-10.2196/63481

## Introduction

### Background

Tension-type headache (TTH) is a neurological disorder characterized by mild to moderate headaches [1]. It is the most common type of headache and second most common health-related complaint among children and adults [2,3], and negatively affects their ability to participate in various activities in school, sports, social, and home settings, especially when the headache becomes chronic and frequent [4]. Many studies have shown that TTH significantly impacts mood, sleep, and liveliness; anxiety or depression is more common in patients with headaches than in those without headaches [5-7].

Traditional Chinese medicine (TCM), which includes Chinese herbal medicine, acupuncture, and massage, plays an increasingly important role in the treatment of TTH [8-13]. In particular, acupuncture is frequently used to treat TTH [14] and has been approved as a supplementary therapy option for TTH by the European Federation of Neurological Societies [15]. Several randomized controlled trials (RCTs) of TCM for TTH have been conducted in China. Although an individual RCT is valuable, pooling data from numerous RCTs can give more substantial evidence to support therapeutic decision-making [16]. The normalization and homogeneity of research outcomes are crucial when pooling data from multiple studies. However, we found the following problems in the selection and reporting of outcomes in RCTs and systematic reviews on the effects of TCM on TTH.

Many studies only reported the effectiveness rate of composite indicators [17], and certain clinical studies failed to include critical or relevant outcomes, such as headache frequency, severity, duration, impact on quality of life, and analgesic use. As a result, these studies were not suitable for secondary analysis [18], which precludes the incorporation of many results into systematic reviews or meta-analyses. This limitation undermines the ability to provide higher-level evidence for clinical practice, thereby diminishing the research’s value and contributing to unnecessary waste. In addition, selective reporting of results may exist in current clinical studies, as researchers are more likely to select statistically significant results, leading to overestimation of outcomes and exaggeration of efficacy [10,13,19]. Outcome measures were poorly defined, with total effectiveness rates inconsistently defined using a variety of concepts [17]. The outcome measures for different studies were heterogeneous, and systematic reviews have shown significant heterogeneity among studies, which is not conducive to intervention comparisons and meta-analysis [10,11,13]. Furthermore, outcomes were developed without considering the opinions of patients and other stakeholders. The International Headache Society Committee proposed an evaluation of outcomes in clinical trials [20]. However, there is still a lack of consensus on the collection and reporting of RCTs of TCM for the treatment of TTH, which limits the comparison and aggregation of data from individual trials and reduces their research value.

Therefore, to address the above problems, it is necessary to develop a core outcome set (COS) for TCM clinical studies (COS-TCM) for TTH. Developing, disseminating, and implementing a COS can address and overcome inconsistencies in outcome selection, measurement, and reporting [21]. Currently, there is no specific COS-TCM for TTH. After searching the Core Outcome Measures in Effectiveness Trials (COMET) database, we identified 1 COS associated with TTH. The International Headache Society Committee proposed an evaluation of TTH outcomes in clinical trials in 1995 (1st edition) [22] and 2010 (2nd edition) [15]. However, the standardized outcome set in COS may be biased toward Western patient populations and lack access to Chinese clinical experts and Chinese patients. There are no COSs available that include outcomes relevant to TCM syndromes. Therefore, developing a COS-TCM for TTH will enhance the quality of evidence from clinical studies of TCM for TTH, promote the translation of clinical research into clinical practice, and provide recommendations for health care decision-making [23,24].

This study was registered in the COMET Initiative (1473) and will be conducted according to the Core Outcome Set-STAndards for Development [25] and Construction of Core Outcome Set of TCM Clinical Trials guidelines [26]. This study protocol referred to the Core Outcome Set-Standardized Protocol Items [27], which are shown in Multimedia Appendix 1.

### Scope and Aim

#### Aim

This study aims to develop a COS for future clinical studies on TCM for the treatment of TTH, in order to improve the use of evidence synthesis by standardizing outcome reporting and guaranteeing that all studies contribute valuable data.

#### Scope

The scope of the COS-TCM encompasses several key areas. It focuses on the health condition of TTH and targets a population of patients with TTH who are aged 18 years and older. The types of interventions included are various TCM therapies, such as herbal medicine decoction, Chinese patent medicine, acupuncture, moxibustion, cupping, massage, Tai Ji, Baduanjin, Qigong, and other nondrug treatments. This scope applies to any type of clinical study.

## Methods

### Design

**Figure 1:**
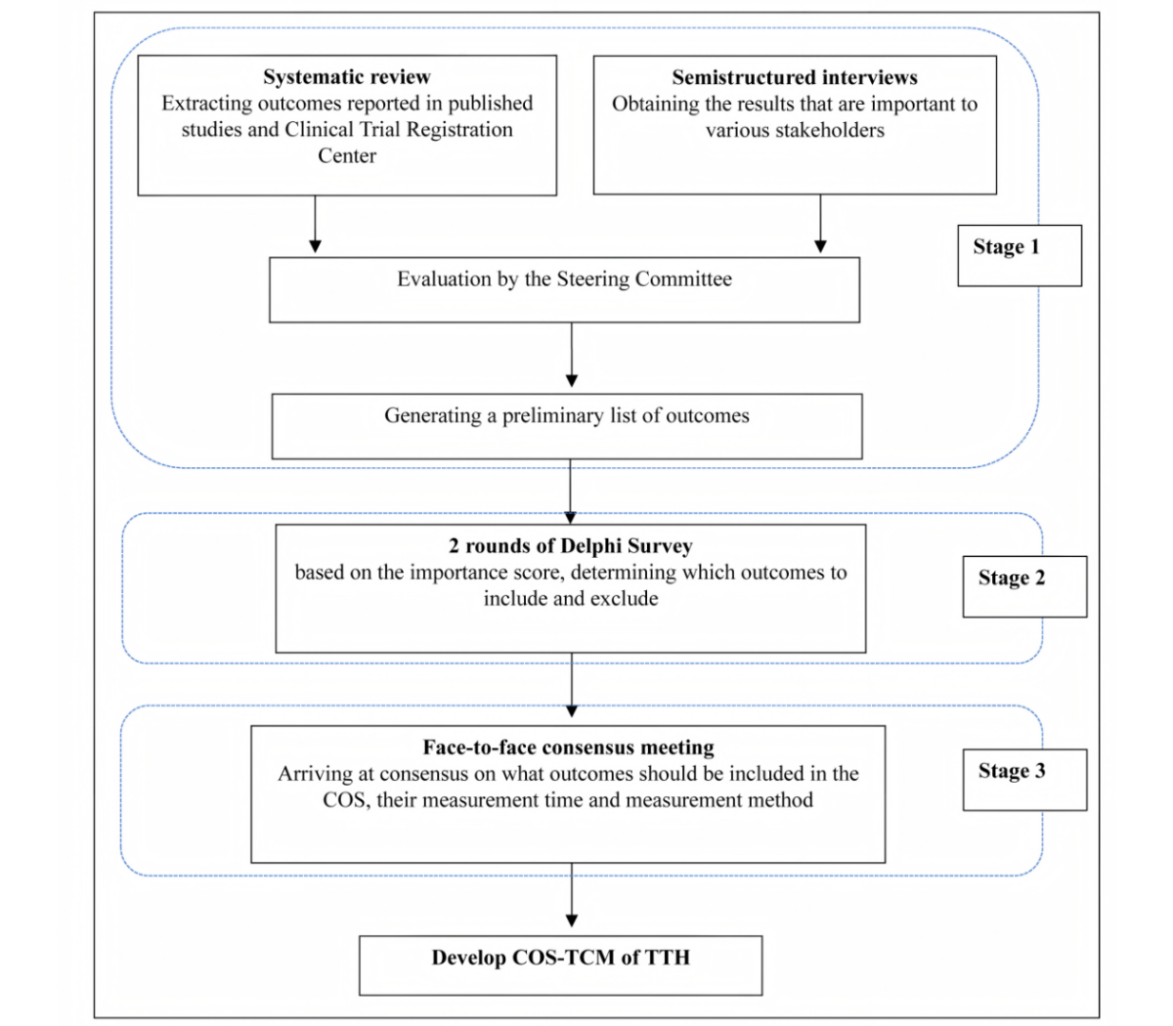
The flowchart of the study core outcome set (COS). TCM: traditional Chinese medicine; TTH: tension-type headache.

The study design is structured in 3 stages, providing a comprehensive framework for developing the COS-TCM. Initially, semistructured interviews and a systematic review will be conducted to identify potential essential outcomes. This will culminate in a preliminary list of outcomes, evaluated and finalized by the steering committee. The second stage involves selecting various stakeholders to participate in a 2-round Delphi survey, aiming to gather diverse opinions on the COS-TCM. Finally, a consensus meeting with key stakeholders will be convened to finalize the COS-TCM. Figure 1 illustrates the flowchart of the study process.

### Steering Group

The steering group will consist of 5 individuals from various scientific fields, including 2 TCM clinical neurology experts, 2 Western medicine clinical neurology experts, and a methodologist. The group will examine and approve the study protocol, determine the preliminary checklist of the reporting outcome set, and take part in a consensus conference to advance the construction of the COS.

### Working Group

The working group will consist of 20 members, including clinicians, methodologists, professors, and graduate students from Xiyuan Hospital, China Academy of Chinese Medical Sciences. The group will be responsible for conducting routine research tasks and related meetings, contacting experts, and seeking advice from the steering committee when disagreements need to be resolved.

### Involvement of Stakeholders

Various stakeholders, including health professionals and patients, will be included in the COS development process. Health professionals will include TCM and Western medicine practitioners who specialize in cerebrovascular disease, methodologists who specialize in evidence-based medicine, and researchers. Core journal editors will select from journals focusing on neurological diseases, such as the *Chinese Journal of Neurology*, *Chinese Archives of Traditional Chinese Medicine*, *Journal of Neurology and Neurorehabilitation*, *Chinese Journal of Evidence-Based Medicine*, and *Global Traditional Chinese Medicine*. Other relevant experts include clinical trial personnel, industry representatives, and policy makers. The study will invite patients with TTH to participate.

### Patient and Public Involvement

Considering that patients’ opinions are significant for the formulation of the COS, this study will involve semistructured interviews, 2 rounds of Delphi survey, and consensus meetings.

### Stage 1: Identification of Potentially Significant Outcomes

#### Step 1: Systematic Review

According to the guidelines of the COMET Initiative [21], a systematic evaluation will be performed to screen the spectrum of critical potential outcomes. Previous COS-TCM studies will be used for systematic review as the research starting point [28-30]. This study will include all clinical studies (regardless of research type) that report TTH outcomes.

##### Search Strategy

PubMed, the Cochrane Library, Embase, Web of Science, SinoMed, Chinese National Knowledge Infrastructure, Chinese Citation Database, China Science Periodical Database, Chinese Clinical Trial Registry, and Clinicaltrials.gov will be systematically searched from inception to January 31, 2024. The search strategy for English databases is shown in Multimedia Appendix 2. The languages will be restricted to English and Chinese.

##### Inclusion Criteria

The inclusion criteria for the study encompass several key aspects. First, the study must be a clinical investigation, which can include randomized controlled studies, case-control studies, cohort trials, or case series. Participants should be patients diagnosed with TTH who are 18 years or older. The diagnosis must be made using any internationally recognized classification, such as those outlined in the International Classification of Headache Disorders, including the first edition (1988), the second edition (2004), and the third edition (2013) by the Headache Classification Committee of the International Headache Society. The intervention under consideration should involve TCM therapies for those included in the treatment group. In addition, if a study primarily investigates secondary TTH as a consequence of conditions such as mental disorders, infections, or endocrine diseases, a thorough review of the full text will be conducted to determine the presence of headache-related outcome measures. Studies that report such outcomes will be eligible for inclusion.

##### Exclusion Criteria

The exclusion criteria encompass several specific aspects. Clinical trials that investigate the outcomes of comorbidities related to TTH, such as nausea caused by TTH, are excluded. In addition, studies primarily focused on assessing pharmacodynamics or pharmacokinetics are not considered. A study involving fewer than 10 cases is also excluded.

##### Data Extraction

A data extraction table was designed and will be used to extract basic study information, including author information, year of publication, country, Western medicine diagnostic standards, type of TTH, TCM syndrome names and diagnostic criteria, interventional measures, study type, conclusions, outcomes and their definitions, time points, and method of outcome measurement. A total of 2 trained reviewers will be responsible for independent study screening and data extraction. Disagreements will be resolved by consulting with a third researcher. If any data is missing, the reviewers will contact the authors of the research by email or telephone to obtain the missing data.

#### Step 2: Semistructured Interview

A systematic review has been conducted to summarize the outcomes of existing clinical studies. However, this mainly represents the researchers’ perspectives. According to the current guideline recommendations of the Core Outcome Set-STAndards for Development [25] and the COMET handbook (version 1.0) ADDIN [31], the opinions of clinicians and patients on the treatment of TTH with TCM can be obtained through semistructured interviews.

##### Participants

In this study, we will use heterogeneous and purposive sampling methods to recruit participants. Purposive sampling is widely used in qualitative and mixed-methods research because it enables researchers to select samples based on specific study objectives and criteria. This approach allows for the selection of more representative samples within a limited timeframe, thus conserving significant time and effort and reducing costs. Specifically, we will recruit patients diagnosed with TTH from the Xiyuan Hospital of the China Academy of Chinese Medical Sciences, as well as clinical doctors from 15 tertiary hospitals nationwide, to ensure the diversity and representativeness of the sample. The criteria for patient selection include age, gender, type of disease, duration of illness, and type of treatment, aiming to capture a broad range of patient experiences and treatment outcomes. For clinical doctors, the selection will focus on geographical diversity and professional background to obtain a wide spectrum of clinical perspectives. We plan to recruit 30 clinical doctors and 30 patients. Although no standardized method exists for determining the sample size for semistructured interviews, previous studies suggest that data saturation typically occurs when the sample size reaches 30 [24,30,32]. By adhering to these criteria and methods, we ensure that the selected sample adequately reflects the diversity and complexity of the research topic, thereby enhancing the quality and reliability of the study findings. Detailed inclusion and exclusion criteria for clinical doctors and patients in the semistructured interviews are provided in Table 1.

**Table 1:** Inclusion and exclusion criteria for clinicians and patients in semistructured interviews.

Category	Inclusion criteria	Exclusion criteria
Clinicians	Senior titles in neurology or TCM^a^, with more than 5 years of work experience. At least a bachelor’s degree. Engage in headache treatment.	None.
Patients	Diagnosed with TTH^b^, no limitations on status. Aged 18 years and older. Receiving TCM treatment. Sign informed consent forms. Capable of reading, understanding, and speaking Chinese or English.	Severe diseases such as heart failure, cerebral infarction, cerebral hemorrhage, and tumors.

^a^TCM: traditional Chinese medicine.

^b^TTH: tension-type headache.

##### Data Collection

Considering previous studies and the characteristics of TTH, we designed a semistructured interview. The development of the interview questions involved a thorough review of existing literature and consultation with experts in the field to ensure content validity. The interview will be conducted by professional researchers in a specific consultation room or office, with face-to-face interactions with participants conducted as often as possible. All interviews will be recorded to facilitate comprehensive data analysis. Before the interview, participants will be informed of the study’s purpose and content, and they will be required to sign an informed consent form.

For patients, the semistructured interview will explore several key areas. Participants will be asked about the duration of their headache experience or how long they have been diagnosed with TTH. They will describe their main symptoms and the treatment they are currently receiving. In addition, they will be queried about their satisfaction with the current treatment and any recommendations they have for its improvement. Further questions will address the areas they would most like to improve and the outcomes they hope to enhance after treatment.

For clinicians, the interview will focus on their professional experience and treatment approaches for TTHs. Clinicians will discuss how long they have worked in their field and their methods for treating tension-type headaches. They will be asked about the outcomes they believe the therapies will enhance for patients. Furthermore, they will identify which outcomes they prioritize in the treatment of TTHs, listing at least 5 indicators they are concerned with.

To evaluate the validity of the interview questions, we conducted a pilot test with a small group of participants and clinicians not involved in the main study. Feedback from this pilot test was used to refine the questions, ensuring clarity and relevance. In addition, an expert panel reviewed the questions to confirm that they accurately capture the dimensions of interest related to TTH.

##### Data Analysis

A total of 2 researchers will independently conduct the data analysis. Disagreements will be resolved through discussions or with the assistance of a third researcher. After sorting out the recorded text, the data will be analyzed using the frame analysis method, including familiarity, identifying thematic frames, indexing, charting, mapping, and interpretation to obtain essential outcomes for patients and clinicians [33].

#### Step 3: Merging and Collating Outcomes

After completing the systematic review and semistructured interviews, the results from these 2 components will be merged. Guided by the COS-TCM standard, 2 researchers will independently collect the outcomes, resolving any disagreements through consultation or with the input of a third researcher. The data collation process begins with importing the extracted indicators into a Microsoft Excel table for sorting. Outcomes are assigned and matched to the corresponding study numbers to facilitate tracing. Following this, the outcomes undergo preliminary sorting, during which duplicates are removed. All study numbers and amounts indicating the results and frequency of application for each outcome are recorded.

Subsequently, the original outcome measures are standardized. For example, the names of outcomes are standardized, and composite results are reduced to a single result without altering the original meaning of the index. Finally, the names and frequencies of all outcomes are counted. The outcome domains are then determined, with the collected outcomes further classified into 7 domains according to the COMET manual [31] and COS-TCM [26] standards: TCM syndromes, symptoms and signs, physical and chemical testing, quality of life, long-term prognosis, economic evaluation, and safety events.

#### Step 4: Generating a Preliminary List of Outcomes

A preliminary list of outcomes will be finalized after evaluation by the steering committee. If the number of results collected in the indicator pool is small (≤100), all the outcomes will be included in the outcome list. If the number exceeds 100, the steering committee will conduct an internal vote on the indicator pool. If 90% of the members do not agree to include an item in the original list, it will be removed. Outcomes added by the steering committee will also be included to form the initial outcome list.

### Stage 2: Delphi Survey

#### Involvement of Stakeholders

Stakeholders, including health care experts and patients with TTH, will be invited to participate. Health care experts include TCM and integrated Chinese and Western medicine practitioners who are engaged in the field of cerebrovascular disease, Western medicine clinicians in the field of cerebrovascular disease, methodologists in the field of evidence-based medicine, researchers, core journal editors, and other relevant experts.

As the project team’s leading unit, the China Academy of Chinese Medical Sciences formed the Encephalopathy Project Team of the TCM Evidence-based Medicine Center with 15 hospitals from 15 provinces. Qualified health care experts from these 15 hospitals will be recruited to participate in the Delphi survey. Patients will be recruited from the Encephalopathy Department of Xiyuan Hospital. The inclusion and exclusion criteria for the health care experts and patients are listed in Table 2.

**Table 2:** Inclusion and exclusion criteria for health professionals and patients in the Delphi survey.

Category	Inclusion criteria	Exclusion criteria
Health professionals	More than 1 year of work experience. Bachelor’s degree or above. Experience working in tertiary hospitals. Published at least one clinical trial on cerebrovascular disease.	None.
Patients	Diagnosed with TTH^a^, no limitations on status. Aged 18 years and older. Receiving TCM^b^ treatment. Sign an informed consent form. Capable of reading, understanding, and speaking Chinese or English.	Patients with severe diseases such as heart failure, cerebral infarction, cerebral hemorrhage, and tumors will be excluded based on medical history, physical examinations, and auxiliary tests.

^a^TTH: tension-type headache.

^b^TCM: traditional Chinese medicine.

#### Sampling Strategy

Since there is no reliable method for estimating the required sample size, the sample size will be determined according to the needs and conditions of the study [34]. Based on the sample size of previous studies and the implementation specification for the Delphi survey in the COS-TCM [26,35,36], we aim to involve 100 stakeholders, including 30 TCM and integrated Chinese and Western medicine clinicians, 15 Western medicine clinicians, 45 patients, 5 researchers, and 10 methodologists. In total, 2 rounds of the Delphi survey will be conducted.

### Round 1 of the Delphi Survey

#### Developing a Questionnaire for Round 1 of the Delphi Survey

A questionnaire for round 1 of the Delphi survey will be formulated based on a preliminary list of outcomes obtained from systematic reviews, semistructured interviews, and evaluation by the steering committee. The questionnaire will consist of 4 parts, including the description of the purpose of the study, personal information of the respondent, evaluation of the importance of outcome indicators, and open questions. Participants will need to provide arguments for the inclusion or exclusion of each outcome in a COS. These arguments can then be summarized and used to develop proposals for voting on the inclusion or exclusion of each outcome in the second round. The open questions will be mainly supplementary to the questions considered necessary by the participants but not included in the questionnaire. To improve the intelligibility of the questionnaire, different terms will be designed for different stakeholder groups. For example, for Western medicine experts, the interpretation of TCM terms can be translated into the Western medicine language in which they are proficient. A general explanation will be added for patients. Stakeholders will be involved in the design of the questionnaire in advance. Delphi survey items commonly use a 9-point critical or relevant outcome to score the importance of outcomes [26,31]. Scores of 1-3 indicate that the outcome is nonessential,” 4-6 indicate that the outcome is “important but not vital,” whereas 7-9 indicate that the outcome is “necessary for inclusion.” If participants are unable to assess the importance of some outcomes, they will be able to select “uncertain.”

#### Process of Round 1 of the Delphi Survey

The first round of the Delphi survey is expected to be completed within 3 weeks. We will email the electronic version of the questionnaire to health care experts. They will be required to complete the questionnaire within 3 weeks and will be reminded by text messages 1 week and 48 hours before the end of the survey. The working group will assess the number of participants at the end of the second week. If the response rate to the Delphi survey (number of respondents/number of invited participants) is less than 70%, the survey will be extended for another 2 weeks. To increase the awareness rate, the questionnaire will be distributed on workdays. We will recruit eligible patients from the Department of Encephalopathy at Xiyuan Hospital. The consulting doctor will be responsible for introducing the content of the questionnaire to the patient and obtaining their consent and signature on the informed consent form. Team members will then hand out questionnaires to patients for immediate completion. We will try our best to answer the patients’ questions.

#### Data Analysis for Delphi Round 1

The working group will gather all submitted questionnaires and calculate the response rate, average score, score distribution among health professionals and patients, and the number of participants from various stakeholder groups for each outcome item. To ensure that the outcomes of Delphi round 1 are fully shown and can be rescored, all outcomes will be retained in subsequent rounds [31,35,36]. Newly added items can be included in Delphi round 2 if the steering committee deems them to be different from the results of Delphi round 1.

### Round 2 of the Delphi Survey

#### Process of Round 2 of the Delphi Survey

Participants who completed the first round of the Delphi survey will be invited to participate in the second round. Each participant will be shown their first-round results, the score distribution of other stakeholders, and a summary of arguments. Based on this feedback, participants will be requested to regrade and score the additional questions in the second round. If the score for an outcome changes significantly between rounds, for example, from “not important” (1-3 points) to “critical” (7-9 points), the rationale for the change will be asked to be mentioned. Participants will also be able to provide suggestions for each survey item.

The Delphi round 2 questionnaires will be distributed like that of round 1 and will be expected to be completed within 3 weeks. For health care professionals, we will provide an electronic version of the questionnaire. A week and 48 hours before the end of the survey, we will send text messages to participants who have not completed the questionnaire. We will recruit eligible patients from the Department of Encephalopathy of Xiyuan Hospital and send the questionnaires to them after obtaining their consent.

#### Data Analysis for Round 2 of the Delphi Survey

After completing the questionnaire, the working group will calculate the response rate, average score, and score distribution for each item. After analyzing all the data, the average scores for the 2 rounds will be compared, and the reasons for score changes will be analyzed to evaluate whether there was attrition. Considering the results of the second round, in combination with the consensus definition, the outcomes will be categorized as “consensus in,” “consensus out,” and “no consensus” (Table 3) [31].

**Table 3:** Definitions of a consensus.

Classification of consensus	Description	Exclusion criteria
Consensus in	Consensus that the outcome should be included in the COS^a^.	≥70% of participants score the outcome as 7-9, and <15% score it as 1–3 in both stakeholder groups.
Consensus out	Consensus that the outcome should not be included in the COS.	≤50% of participants score the outcome as 7-9 in both stakeholder groups.
No consensus	Uncertainty about the importance of the outcome.	Anything else.

^a^COS: core outcome set.

### Stage 3: Consensus Meeting

#### Stakeholder Selection

After the 2 rounds of the Delphi survey, we will conduct a consensus meeting. To ensure the quality of the meeting and enhance the credibility of the results, we will invite representatives of various interest groups who have completed all Delphi surveys, steering committee members, and other representative senior experts from various stakeholder groups, regardless of their participation in the previous research process. At the same time, senior clinical experts in the field of TCM, especially academicians, TCM masters, nationally famous TCM practitioners, and leaders of academic groups, will be invited. Patients who participated in both rounds of the Delphi survey will be invited to the consensus conference. The inclusion and exclusion criteria for health professionals who will participate in the consensus meetings are listed in Textbox 1.

The first and the second must be qualified, while the third and the fourth can be qualified by any one of them.

**The inclusion and exclusion criteria for health care professionals who will participate in the consensus meeting.**

Inclusion criteria:

- Master’s degree or above, with more than 10 years of work experience.
- Clinicians with experience working at tertiary hospitals and at least in the position of associate chief physician.
- Participated in or hosted clinical research projects related to headache.
- Familiarity with evidence-based medicine and methodological research.

Exclusion criteria:

- None.

#### Sampling Strategy

According to the current guideline recommendations of COS-TCM standards [26], we will invite 25 stakeholders from all over the country, including 9 TCM experts, 5 Western medicine experts, 3 researchers, 5 methodologists, and 3 patients, to participate in the consensus meeting.

#### Consensus Meeting Process

The consensus meeting to determine the final COS will be face to face, preferably in Beijing. In exceptional cases, a network video conference will be held. The meeting will last for 2 days.

We will report on our previous work, including the preliminary list of outcomes generated using a systematic review and semistructured interviews and the results of the 2 rounds of Delphi. We will focus on reporting the outcomes of the second round, with outcomes categorized as “consensus in” from all stakeholder groups being prioritized for inclusion in the COS, and outcomes categorized as “consensus out” being excluded. The outcomes categorized as “no consensus” will be discussed at the meeting, and all participants will rate their importance using a 9-point Likert scale. The final COS will be developed according to the consensus definitions [5]. If some outcomes are still considered “no consensus” after 2 rounds of grading, the steering group will determine their inclusion in the final COS.

After formulating the final COS, the measurement method for the outcome index will be determined. The questionnaire will be designed based on the results of the system evaluation. Participants will discuss and vote on the measurement time and methods of the outcome indicators. For the measurement time and method of each outcome indicator, we will select the indicator with the highest percentage recommended in the consensus meeting.

### Ethical Considerations

This study received approval from the Ethics Committee of Xiyuan Hospital, China Academy of Chinese Medical Sciences (approval number 2021XLA003-1). We followed the committee’s ethical guidelines rigorously to ensure compliance and safeguard participant rights. All participants provided informed consent after receiving comprehensive information about the study, and they were assured that their involvement was voluntary, with the option to withdraw at any time without repercussions. We are dedicated to maintaining participant privacy and confidentiality; all data were anonymized and securely stored, accessible only to the research team. The findings will be presented in a way that does not reveal individual identities. There are no conflicts of interest in this study, and while participants did not receive financial compensation, we extend our heartfelt thanks for their contributions. Data for the systematic review were sourced from publicly available literature, in line with ethical standards for data sharing. The outcomes from the Delphi survey and consensus meeting will contribute to the development of the COS-TCM and will be reported without identifying individual participants.

## Results

The protocol for this study has been registered in PROSPERO. The literature search has been completed, and the analysis of the systematic review results is currently under review. The findings will be published once the review process is finalized. A total of 19,033 articles were retrieved from 8 databases. After merging Chinese and English records and removing duplicates, 8074 duplicate articles were identified. Through title and abstract screening, 8844 articles were excluded for irrelevance, and 45 articles were excluded for being in languages other than Chinese or English. Further full-text review led to the exclusion of 672 articles for irrelevance, 67 articles due to inaccessible full texts, and 5 articles due to single-author, noncore publications. This process resulted in 1335 articles related to TCM.

Among these, there were 52 guidelines and clinical pathways, 102 systematic reviews and meta-analyses, 590 RCTs (including 177 on acupuncture, 293 on Chinese herbal medicine, and 120 on other therapies), 66 nonrandomized controlled trials, 1 cohort study, 4 case-control studies, 27 protocol registrations, 113 case series, 28 case reports, 21 cross-sectional studies, 226 review articles, 91 expert opinions, and 14 animal experiments. After the abstract screening, 650 articles were excluded, and 24 articles were excluded after a full-text review due to a lack of reported outcomes. Finally, 626 articles were included for further analysis. The PRISMA-P (Preferred Reporting Items for Systematic Review and Meta-Analysis Protocols) checklist is provided in Multimedia Appendix 3 of the initial draft. The Delphi survey involving stakeholders is scheduled to begin on November 30, 2024, and will be followed by a face-to-face consensus conference on February 1, 2025.

Upon completion of the development of the COS, the study findings will be reported in adherence to the COS-STAR (Core Outcome Set–STAndards for Reporting) statement [37]. This study will be disseminated through publication in an international journal and presentation at national and international conferences focused on TTH to promote the adoption of the Clinical Outcome Scale. The COS findings will be distributed to all participants by email or courier to support its clinical use.

## Discussion

### Expected Findings

Given the differences among various headache types, it is essential to specify the headache type of study participants in the clinical research design to ensure study accuracy. TTH represents the most common primary headaches and are characterized by pressing or tightening (nonpulsating) sensations on both sides of the head, are of mild or moderate intensity, and are not exacerbated by activity [38]. The current research on COS for headaches mainly focuses on migraine, such as the guidance on the design of outcome indicators for clinical trials of medications, patient-valued indicators, and identification of meaningful migraine outcome measures [38-40]. Compared with migraine, TTH generally receives less attention but has a substantial impact on individuals and society, compromising the quality of life in terms of work, study, and sleep and causing a social burden that cannot be overlooked [2,41-43]. Currently, only 1 published COS is available for TTH, which was initially published in 1995 [22] and updated in 2010 [20]. The clinical research of TCM on TTH have been increasing over the past 10 years [17]. However, there is no COS for TCM clinical research on the treatment of TTH. TCM has a unique diagnosis and treatment mode, and there are significant differences between the evaluation outcomes of TCM and Western medicine [44]. Thus, developing a COS for clinical studies on TCM for the treatment of TTH is necessary.

This study was designed according to standard procedures and developed in 3 stages [25,26,31], with each stage conducted under the guidance of the steering group. In designing the COS, special attention is given to several key aspects. In the systematic review, we aim to include the intervention methods of TCM as comprehensively as possible to ensure the thoroughness of literature retrieval. Given the prevalence of nonstandard outcomes in clinical trials of TCM, we will standardize the outcomes obtained from systematic reviews and semistructured interviews. This includes standardizing the names of outcomes, dividing composite outcomes into single outcomes, and classifying outcomes into specific domains to ensure the standardization of COS [37].

Given the limited number of clinical RCTs for TCM treatment of TTHs, we have included observational studies to enrich the core outcome set, increase the diversity of outcomes, and enhance the robustness of our core outcome set. This approach ensures a more comprehensive evaluation of the effectiveness and safety of TCM treatments. To address the differences between these 2 types of studies, we plan to establish different core outcome sets for experimental (RCT) and observational studies. For example, in RCTs, we may focus on factors such as treatment duration and cost, whereas in observational studies, we may emphasize long-term outcomes and the impact on quality of life.

In this study, we included various TCM treatment methods to evaluate their comprehensive impact on tension-type headaches. This diversity helps capture the broad efficacy and patient responses to different treatment methods. However, it also introduces complexity in interpreting the results. We believe this approach is beneficial. First, the inclusion of treatment diversity allows us to assess the overall impact of TCM treatments on tension-type headaches. This method enables us to observe potential synergistic effects and individual differences among different treatment methods, providing more comprehensive guidance for clinical practice. Second, to address the variability of treatment methods, we used standardized outcome measurement tools in the study design. These tools ensure the comparability of results between different treatment methods. In addition, we used stratified analysis and subgroup analysis to handle data heterogeneity, ensuring the robustness of the results. It must be acknowledged that including various TCM treatment methods may introduce a certain degree of heterogeneity, which could affect the interpretation of results. Therefore, we used mixed-effects models in the analysis to adjust for potential differences between different treatment methods.

The study will also comprehensively cover different stakeholders in the Delphi survey and consensus meetings. This includes TCM and Western medicine practitioners, methodologists, researchers, core journal editors, and other relevant experts, as well as patients with TTH, to ensure the representativeness and authority of the COS. In designing the questionnaire for the Delphi survey, we will focus on improving its intelligibility. For instance, TCM terminology will be translated into the language of Western medicine for specialists, while patients will receive a general explanation. The questionnaire distribution will be tailored to the characteristics of stakeholders: healthcare experts will receive a combination of email and electronic questionnaires for efficiency and better data statistics, while patients will complete the questionnaire face to face with a doctor to enhance compliance and understanding. These measures are intended to ensure a higher response rate.

### Conclusion

There is a lack of COS for TCM treatment of TTH; therefore, developing one is essential. This study will solve problems posed by nonstandard outcome indicators and limited measurement time, maximize the value of individual trial data, and provide high-quality research evidence for treating TTH with TCM.

## Appendix

Multimedia Appendix 1 The COS-STAP (Core Outcome Set-STAndardised Protocol Items) Statement.

Multimedia Appendix 2 The search strategy of English databases.

Multimedia Appendix 3 PRISMA (Preferred Reporting Items for Systematic Reviews and Meta-Analyses) checklist.

## Abbreviations

COMET: Core Outcome Measures in Effectiveness Trials
COS: core outcome set
COS-STAR: Core Outcome Set–STAndards for Reporting
COS-TCM: core outcome set for TCM clinical studies
PRISMA-P: Preferred Reporting Items for Systematic Review and Meta-Analysis Protocols
RCT: randomized controlled trial
TCM: traditional Chinese medicine
TTH: tension-type headache
