# Supplementary material for: Core Outcome Set Development for Tension-Type Headache Treatment Using Traditional Chinese Medicine: Protocol for a Delphi Consensus Study
Source: JMIR Res Protoc. 2025 Feb 5;14:e63481. doi: 10.2196/63481 (PMC11840383; doi:10.2196/63481)
Supplement: Multimedia Appendix 2 [file resprot_v14i1e63481_app2.docx]

The search strategy of English databases

1. **Pubmed 2024/08/10**

**#1** "Tension-Type Headache"[Mesh]

**#2** Medicine, chinese traditional[MeSH Terms]

**#3** Herbal Medicine[MeSH Terms]

**#4** Medicine, Traditional[MeSH Terms]

**#5** Complementary Therapies[MeSH Terms]

**#6** Acupuncture[MeSH Terms]

**#7** Moxibustion[MeSH Terms]

**#8** Massage[MeSH Terms]

**#9** Cupping Therapy[MeSH Terms]

**#10** Qigong[MeSH Terms]

**#11** Tai Ji[MeSH Terms]

**#12** ((((((((((((((((((((((((((((Tension-Type Headache[Title/Abstract]) OR (Headache, Tension-Type[Title/Abstract])) OR (Headaches, Tension-Type[Title/Abstract])) OR (Tension Type Headache[Title/Abstract])) OR (Tension-Type Headaches[Title/Abstract])) OR (Idiopathic Headache[Title/Abstract])) OR (Headache, Idiopathic[Title/Abstract])) OR (Headaches, Idiopathic[Title/Abstract])) OR (Idiopathic Headaches[Title/Abstract])) OR (Stress Headache[Title/Abstract])) OR (Headache, Stress[Title/Abstract])) OR (Headaches, Stress[Title/Abstract])) OR (Stress Headaches[Title/Abstract])) OR (Tension Headache[Title/Abstract])) OR (Headache, Tension[Title/Abstract])) OR (Headaches, Tension[Title/Abstract])) OR (Tension Headaches[Title/Abstract])) OR (Psychogenic Headache[Title/Abstract])) OR (Headache, Psychogenic[Title/Abstract])) OR (Headaches, Psychogenic[Title/Abstract])) OR (Psychogenic Headaches[Title/Abstract])) OR (Tension-Vascular Headache[Title/Abstract])) OR (Headache, Tension-Vascular[Title/Abstract])) OR (Headaches, Tension-Vascular[Title/Abstract])) OR (Tension Vascular Headache[Title/Abstract])) OR (Tension-Vascular Headaches[Title/Abstract])) OR (New daily persistent headache[Title/Abstract])) OR (Contraction headache[Title/Abstract])

**#13** ((((((((((((((Medicine, Chinese Traditional[Title/Abstract]) OR (Traditional Chinese Medicine[Title/Abstract])) OR (Chung I Hsueh[Title/Abstract])) OR (Hsueh, Chung I[Title/Abstract])) OR (Traditional Medicine, Chinese[Title/Abstract])) OR (Zhong Yi Xue[Title/Abstract])) OR (Chinese Traditional Medicine[Title/Abstract])) OR (Chinese Medicine, Traditional[Title/Abstract])) OR (Traditional Tongue Diagnosis[Title/Abstract])) OR (Tongue Diagnoses, Traditional[Title/Abstract])) OR (Tongue Diagnosis, Traditional[Title/Abstract])) OR (Traditional Tongue Diagnoses[Title/Abstract])) OR (Traditional Tongue Assessment[Title/Abstract])) OR (Tongue Assessment, Traditional[Title/Abstract])) OR (Traditional Tongue Assessments[Title/Abstract])

**#14** (((((((((((Herbal Medicine[Title/Abstract]) OR (Medicine, Herbal[Title/Abstract])) OR (Hawaiian Herbal Medicine[Title/Abstract])) OR (Hawaiian Herbal Medicines[Title/Abstract])) OR (Herbal Medicine, Hawaiian[Title/Abstract])) OR (Herbal Medicines, Hawaiian[Title/Abstract])) OR (Medicine, Hawaiian Herbal[Title/Abstract])) OR (Medicines, Hawaiian Herbal[Title/Abstract])) OR (La'au Lapa'au[Title/Abstract])) OR (Laau Lapaau[Title/Abstract])) OR (La au Lapa au[Title/Abstract])) OR (Herbalism[Title/Abstract])

**#15** ((((((((((((((((Medicine, Traditional[Title/Abstract]) OR (Traditional Medicine[Title/Abstract])) OR (Home Remedies[Title/Abstract])) OR (Home Remedy[Title/Abstract])) OR (Remedies, Homeziy[Title/Abstract])) OR (Remedy, Home[Title/Abstract])) OR (Medicine, Primitive[Title/Abstract])) OR (Primitive Medicine[Title/Abstract])) OR (Medicine, Folk[Title/Abstract])) OR (Folk Medicine[Title/Abstract])) OR (Medicine, Indigenous[Title/Abstract])) OR (Indigenous Medicine[Title/Abstract])) OR (Folk Remedies[Title/Abstract])) OR (Folk Remedy[Title/Abstract])) OR (Remedies, Folk[Title/Abstract])) OR (Remedy, Folk[Title/Abstract])) OR (Ethnomedicine[Title/Abstract])

**#16** ((((((((((((((((((((((((((Acupuncture[Title/Abstract]) OR (Pharmacopuncture[Title/Abstract])) OR (Acupuncture Treatment[Title/Abstract])) OR (Acupuncture Treatments[Title/Abstract])) OR (Treatment, Acupuncture[Title/Abstract])) OR (Therapy, Acupuncture[Title/Abstract])) OR (Pharmacoacupuncture Treatment[Title/Abstract])) OR (Treatment, Pharmacoacupuncture[Title/Abstract])) OR (Pharmacoacupuncture Therapy[Title/Abstract])) OR (Therapy, Pharmacoacupuncture[Title/Abstract])) OR (Acupotomy[Title/Abstract])) OR (Acupotomies[Title/Abstract])) OR (Acupunctures, Ear[Title/Abstract])) OR (Ear Acupunctures[Title/Abstract])) OR (Auricular Acupuncture[Title/Abstract])) OR (Ear Acupuncture[Title/Abstract])) OR (Acupuncture, Auricular[Title/Abstract])) OR (Acupunctures, Auricular[Title/Abstract])) OR (Auricular Acupunctures[Title/Abstract])) OR (Acupuncture Point[Title/Abstract])) OR (Point, Acupuncture[Title/Abstract])) OR (Points, Acupuncture[Title/Abstract])) OR (Acupoints[Title/Abstract])) OR (Acupoint[Title/Abstract])) OR (Analgesia, Acupuncture[Title/Abstract])) OR (Acupuncture Anesthesia[Title/Abstract])) OR (Anesthesia, Acupuncture[Title/Abstract])

**#17** (Moxibustion[Title/Abstract]) OR (Moxabustion[Title/Abstract])

**#18** ((((((((Massage[Title/Abstract]) OR (Zone Therapy[Title/Abstract])) OR (Therapies, Zone[Title/Abstract])) OR (Zone Therapies[Title/Abstract])) OR (Therapy, Zone[Title/Abstract])) OR (Massage Therapy[Title/Abstract])) OR (Massage Therapies[Title/Abstract])) OR (Therapies, Massage[Title/Abstract])) OR (Therapy, Massage[Title/Abstract])

**#19** (((((Cupping Therapy[Title/Abstract]) OR (Cupping Therapies[Title/Abstract])) OR (Therapy, Cupping[Title/Abstract])) OR (Cupping Treatment[Title/Abstract])) OR (Cupping Treatments[Title/Abstract])) OR (Treatment, Cupping[Title/Abstract])

**#20** ((Qigong[Title/Abstract]) OR (Qi Gong[Title/Abstract])) OR (Ch'i Kung[Title/Abstract])

**#21** ((((((((((Tai Ji[Title/Abstract]) OR (Tai-ji[Title/Abstract])) OR (Tai Chi[Title/Abstract])) OR (Chi, Tai[Title/Abstract])) OR (Tai Ji Quan[Title/Abstract])) OR (Ji Quan, Tai[Title/Abstract])) OR (Quan, Tai Ji[Title/Abstract])) OR (Taiji[Title/Abstract])) OR (Taijiquan[Title/Abstract])) OR (T'ai Chi[Title/Abstract])) OR (Tai Chi Chuan[Title/Abstract])

**#22** ((baguan[Title/Abstract]) OR (baduanjin[Title/Abstract])) OR (tuina[Title/Abstract])

**#23** ((((((((((Therapies, Complementary[Title/Abstract])) OR (Therapy, Complementary[Title/Abstract])) OR (Complementary Medicine[Title/Abstract])) OR (Medicine, Complementary[Title/Abstract])) OR (Alternative Medicine[Title/Abstract])) OR (Medicine, Alternative[Title/Abstract])) OR (Alternative Therapies[Title/Abstract])) OR (Therapies, Alternative[Title/Abstract])) OR (Therapy, Alternative[Title/Abstract])) OR (Complementary Therapies[Title/Abstract])

**#24** (((((((((((Therapies, Complementary[Title/Abstract])) OR (Therapy, Complementary[Title/Abstract])) OR (Complementary Medicine[Title/Abstract])) OR (Medicine, Complementary[Title/Abstract])) OR (Alternative Medicine[Title/Abstract])) OR (Medicine, Alternative[Title/Abstract])) OR (Alternative Therapies[Title/Abstract])) OR (Therapies, Alternative[Title/Abstract])) OR (Therapy, Alternative[Title/Abstract])) OR (Complementary Therapies[Title/Abstract])) OR (Complementary Therapies[MeSH Terms])

**#25** (((((((((((Tai Ji[Title/Abstract]) OR (Tai-ji[Title/Abstract])) OR (Tai Chi[Title/Abstract])) OR (Chi, Tai[Title/Abstract])) OR (Tai Ji Quan[Title/Abstract])) OR (Ji Quan, Tai[Title/Abstract])) OR (Quan, Tai Ji[Title/Abstract])) OR (Taiji[Title/Abstract])) OR (Taijiquan[Title/Abstract])) OR (T'ai Chi[Title/Abstract])) OR (Tai Chi Chuan[Title/Abstract])) OR (Tai Ji[MeSH Terms])

**#26** (((Qigong[Title/Abstract]) OR (Qi Gong[Title/Abstract])) OR (Ch'i Kung[Title/Abstract])) OR (Qigong[MeSH Terms])

**#27** ((((((Cupping Therapy[Title/Abstract]) OR (Cupping Therapies[Title/Abstract])) OR (Therapy, Cupping[Title/Abstract])) OR (Cupping Treatment[Title/Abstract])) OR (Cupping Treatments[Title/Abstract])) OR (Treatment, Cupping[Title/Abstract])) OR (Cupping Therapy[MeSH Terms])

**#28** (((((((((Massage[Title/Abstract]) OR (Zone Therapy[Title/Abstract])) OR (Therapies, Zone[Title/Abstract])) OR (Zone Therapies[Title/Abstract])) OR (Therapy, Zone[Title/Abstract])) OR (Massage Therapy[Title/Abstract])) OR (Massage Therapies[Title/Abstract])) OR (Therapies, Massage[Title/Abstract])) OR (Therapy, Massage[Title/Abstract])) OR (Massage[MeSH Terms])

**#29** ((Moxibustion[Title/Abstract]) OR (Moxabustion[Title/Abstract])) OR (Moxibustion[MeSH Terms])

**#30** (((((((((((((((((((((((((((Acupuncture[Title/Abstract]) OR (Pharmacopuncture[Title/Abstract])) OR (Acupuncture Treatment[Title/Abstract])) OR (Acupuncture Treatments[Title/Abstract])) OR (Treatment, Acupuncture[Title/Abstract])) OR (Therapy, Acupuncture[Title/Abstract])) OR (Pharmacoacupuncture Treatment[Title/Abstract])) OR (Treatment, Pharmacoacupuncture[Title/Abstract])) OR (Pharmacoacupuncture Therapy[Title/Abstract])) OR (Therapy, Pharmacoacupuncture[Title/Abstract])) OR (Acupotomy[Title/Abstract])) OR (Acupotomies[Title/Abstract])) OR (Acupunctures, Ear[Title/Abstract])) OR (Ear Acupunctures[Title/Abstract])) OR (Auricular Acupuncture[Title/Abstract])) OR (Ear Acupuncture[Title/Abstract])) OR (Acupuncture, Auricular[Title/Abstract])) OR (Acupunctures, Auricular[Title/Abstract])) OR (Auricular Acupunctures[Title/Abstract])) OR (Acupuncture Point[Title/Abstract])) OR (Point, Acupuncture[Title/Abstract])) OR (Points, Acupuncture[Title/Abstract])) OR (Acupoints[Title/Abstract])) OR (Acupoint[Title/Abstract])) OR (Analgesia, Acupuncture[Title/Abstract])) OR (Acupuncture Anesthesia[Title/Abstract])) OR (Anesthesia, Acupuncture[Title/Abstract])) OR (Acupuncture[MeSH Terms])

**#31** (((((((((((((((((Medicine, Traditional[Title/Abstract]) OR (Traditional Medicine[Title/Abstract])) OR (Home Remedies[Title/Abstract])) OR (Home Remedy[Title/Abstract])) OR (Remedies, Homeziy[Title/Abstract])) OR (Remedy, Home[Title/Abstract])) OR (Medicine, Primitive[Title/Abstract])) OR (Primitive Medicine[Title/Abstract])) OR (Medicine, Folk[Title/Abstract])) OR (Folk Medicine[Title/Abstract])) OR (Medicine, Indigenous[Title/Abstract])) OR (Indigenous Medicine[Title/Abstract])) OR (Folk Remedies[Title/Abstract])) OR (Folk Remedy[Title/Abstract])) OR (Remedies, Folk[Title/Abstract])) OR (Remedy, Folk[Title/Abstract])) OR (Ethnomedicine[Title/Abstract])) OR (Medicine, Traditional[MeSH Terms])

**#32** ((((((((((((Herbal Medicine[Title/Abstract]) OR (Medicine, Herbal[Title/Abstract])) OR (Hawaiian Herbal Medicine[Title/Abstract])) OR (Hawaiian Herbal Medicines[Title/Abstract])) OR (Herbal Medicine, Hawaiian[Title/Abstract])) OR (Herbal Medicines, Hawaiian[Title/Abstract])) OR (Medicine, Hawaiian Herbal[Title/Abstract])) OR (Medicines, Hawaiian Herbal[Title/Abstract])) OR (La'au Lapa'au[Title/Abstract])) OR (Laau Lapaau[Title/Abstract])) OR (La au Lapa au[Title/Abstract])) OR (Herbalism[Title/Abstract])) OR (Herbal Medicine[MeSH Terms])

**#33** (((((((((((((((Medicine, Chinese Traditional[Title/Abstract]) OR (Traditional Chinese Medicine[Title/Abstract])) OR (Chung I Hsueh[Title/Abstract])) OR (Hsueh, Chung I[Title/Abstract])) OR (Traditional Medicine, Chinese[Title/Abstract])) OR (Zhong Yi Xue[Title/Abstract])) OR (Chinese Traditional Medicine[Title/Abstract])) OR (Chinese Medicine, Traditional[Title/Abstract])) OR (Traditional Tongue Diagnosis[Title/Abstract])) OR (Tongue Diagnoses, Traditional[Title/Abstract])) OR (Tongue Diagnosis, Traditional[Title/Abstract])) OR (Traditional Tongue Diagnoses[Title/Abstract])) OR (Traditional Tongue Assessment[Title/Abstract])) OR (Tongue Assessment, Traditional[Title/Abstract])) OR (Traditional Tongue Assessments[Title/Abstract])) OR (Medicine, chinese traditional[MeSH Terms])

**#34** (((((((((((((((((((((((((((((Tension-Type Headache[Title/Abstract]) OR (Headache, Tension-Type[Title/Abstract])) OR (Headaches, Tension-Type[Title/Abstract])) OR (Tension Type Headache[Title/Abstract])) OR (Tension-Type Headaches[Title/Abstract])) OR (Idiopathic Headache[Title/Abstract])) OR (Headache, Idiopathic[Title/Abstract])) OR (Headaches, Idiopathic[Title/Abstract])) OR (Idiopathic Headaches[Title/Abstract])) OR (Stress Headache[Title/Abstract])) OR (Headache, Stress[Title/Abstract])) OR (Headaches, Stress[Title/Abstract])) OR (Stress Headaches[Title/Abstract])) OR (Tension Headache[Title/Abstract])) OR (Headache, Tension[Title/Abstract])) OR (Headaches, Tension[Title/Abstract])) OR (Tension Headaches[Title/Abstract])) OR (Psychogenic Headache[Title/Abstract])) OR (Headache, Psychogenic[Title/Abstract])) OR (Headaches, Psychogenic[Title/Abstract])) OR (Psychogenic Headaches[Title/Abstract])) OR (Tension-Vascular Headache[Title/Abstract])) OR (Headache, Tension-Vascular[Title/Abstract])) OR (Headaches, Tension-Vascular[Title/Abstract])) OR (Tension Vascular Headache[Title/Abstract])) OR (Tension-Vascular Headaches[Title/Abstract])) OR (New daily persistent headache[Title/Abstract])) OR (Contraction headache[Title/Abstract])) OR ("Tension-Type Headache"[Mesh])

**#35** ((((((((((((((((((((((((Medicine, Chinese Traditional[Title/Abstract]) OR (Traditional Chinese Medicine[Title/Abstract])) OR (Chung I Hsueh[Title/Abstract])) OR (Hsueh, Chung I[Title/Abstract])) OR (Traditional Medicine, Chinese[Title/Abstract])) OR (Zhong Yi Xue[Title/Abstract])) OR (Chinese Traditional Medicine[Title/Abstract])) OR (Chinese Medicine, Traditional[Title/Abstract])) OR (Traditional Tongue Diagnosis[Title/Abstract])) OR (Tongue Diagnoses, Traditional[Title/Abstract])) OR (Tongue Diagnosis, Traditional[Title/Abstract])) OR (Traditional Tongue Diagnoses[Title/Abstract])) OR (Traditional Tongue Assessment[Title/Abstract])) OR (Tongue Assessment, Traditional[Title/Abstract])) OR (Traditional Tongue Assessments[Title/Abstract])) OR (Medicine, chinese traditional[MeSH Terms])) OR (((((((((((((Herbal Medicine[Title/Abstract]) OR (Medicine, Herbal[Title/Abstract])) OR (Hawaiian Herbal Medicine[Title/Abstract])) OR (Hawaiian Herbal Medicines[Title/Abstract])) OR (Herbal Medicine, Hawaiian[Title/Abstract])) OR (Herbal Medicines, Hawaiian[Title/Abstract])) OR (Medicine, Hawaiian Herbal[Title/Abstract])) OR (Medicines, Hawaiian Herbal[Title/Abstract])) OR (La'au Lapa'au[Title/Abstract])) OR (Laau Lapaau[Title/Abstract])) OR (La au Lapa au[Title/Abstract])) OR (Herbalism[Title/Abstract])) OR (Herbal Medicine[MeSH Terms]))) OR ((((((((((((((((((Medicine, Traditional[Title/Abstract]) OR (Traditional Medicine[Title/Abstract])) OR (Home Remedies[Title/Abstract])) OR (Home Remedy[Title/Abstract])) OR (Remedies, Homeziy[Title/Abstract])) OR (Remedy, Home[Title/Abstract])) OR (Medicine, Primitive[Title/Abstract])) OR (Primitive Medicine[Title/Abstract])) OR (Medicine, Folk[Title/Abstract])) OR (Folk Medicine[Title/Abstract])) OR (Medicine, Indigenous[Title/Abstract])) OR (Indigenous Medicine[Title/Abstract])) OR (Folk Remedies[Title/Abstract])) OR (Folk Remedy[Title/Abstract])) OR (Remedies, Folk[Title/Abstract])) OR (Remedy, Folk[Title/Abstract])) OR (Ethnomedicine[Title/Abstract])) OR (Medicine, Traditional[MeSH Terms]))) OR ((((((((((((((((((((((((((((Acupuncture[Title/Abstract]) OR (Pharmacopuncture[Title/Abstract])) OR (Acupuncture Treatment[Title/Abstract])) OR (Acupuncture Treatments[Title/Abstract])) OR (Treatment, Acupuncture[Title/Abstract])) OR (Therapy, Acupuncture[Title/Abstract])) OR (Pharmacoacupuncture Treatment[Title/Abstract])) OR (Treatment, Pharmacoacupuncture[Title/Abstract])) OR (Pharmacoacupuncture Therapy[Title/Abstract])) OR (Therapy, Pharmacoacupuncture[Title/Abstract])) OR (Acupotomy[Title/Abstract])) OR (Acupotomies[Title/Abstract])) OR (Acupunctures, Ear[Title/Abstract])) OR (Ear Acupunctures[Title/Abstract])) OR (Auricular Acupuncture[Title/Abstract])) OR (Ear Acupuncture[Title/Abstract])) OR (Acupuncture, Auricular[Title/Abstract])) OR (Acupunctures, Auricular[Title/Abstract])) OR (Auricular Acupunctures[Title/Abstract])) OR (Acupuncture Point[Title/Abstract])) OR (Point, Acupuncture[Title/Abstract])) OR (Points, Acupuncture[Title/Abstract])) OR (Acupoints[Title/Abstract])) OR (Acupoint[Title/Abstract])) OR (Analgesia, Acupuncture[Title/Abstract])) OR (Acupuncture Anesthesia[Title/Abstract])) OR (Anesthesia, Acupuncture[Title/Abstract])) OR (Acupuncture[MeSH Terms]))) OR (((Moxibustion[Title/Abstract]) OR (Moxabustion[Title/Abstract])) OR (Moxibustion[MeSH Terms]))) OR ((((((((((Massage[Title/Abstract]) OR (Zone Therapy[Title/Abstract])) OR (Therapies, Zone[Title/Abstract])) OR (Zone Therapies[Title/Abstract])) OR (Therapy, Zone[Title/Abstract])) OR (Massage Therapy[Title/Abstract])) OR (Massage Therapies[Title/Abstract])) OR (Therapies, Massage[Title/Abstract])) OR (Therapy, Massage[Title/Abstract])) OR (Massage[MeSH Terms]))) OR (((((((Cupping Therapy[Title/Abstract]) OR (Cupping Therapies[Title/Abstract])) OR (Therapy, Cupping[Title/Abstract])) OR (Cupping Treatment[Title/Abstract])) OR (Cupping Treatments[Title/Abstract])) OR (Treatment, Cupping[Title/Abstract])) OR (Cupping Therapy[MeSH Terms]))) OR ((((Qigong[Title/Abstract]) OR (Qi Gong[Title/Abstract])) OR (Ch'i Kung[Title/Abstract])) OR (Qigong[MeSH Terms]))) OR ((((((((((((Tai Ji[Title/Abstract]) OR (Tai-ji[Title/Abstract])) OR (Tai Chi[Title/Abstract])) OR (Chi, Tai[Title/Abstract])) OR (Tai Ji Quan[Title/Abstract])) OR (Ji Quan, Tai[Title/Abstract])) OR (Quan, Tai Ji[Title/Abstract])) OR (Taiji[Title/Abstract])) OR (Taijiquan[Title/Abstract])) OR (T'ai Chi[Title/Abstract])) OR (Tai Chi Chuan[Title/Abstract])) OR (Tai Ji[MeSH Terms]))) OR ((((((((((((Therapies, Complementary[Title/Abstract])) OR (Therapy, Complementary[Title/Abstract])) OR (Complementary Medicine[Title/Abstract])) OR (Medicine, Complementary[Title/Abstract])) OR (Alternative Medicine[Title/Abstract])) OR (Medicine, Alternative[Title/Abstract])) OR (Alternative Therapies[Title/Abstract])) OR (Therapies, Alternative[Title/Abstract])) OR (Therapy, Alternative[Title/Abstract])) OR (Complementary Therapies[Title/Abstract])) OR (Complementary Therapies[MeSH Terms]))

**#36** (((((((((((((((((((((((((Medicine, Chinese Traditional[Title/Abstract]) OR (Traditional Chinese Medicine[Title/Abstract])) OR (Chung I Hsueh[Title/Abstract])) OR (Hsueh, Chung I[Title/Abstract])) OR (Traditional Medicine, Chinese[Title/Abstract])) OR (Zhong Yi Xue[Title/Abstract])) OR (Chinese Traditional Medicine[Title/Abstract])) OR (Chinese Medicine, Traditional[Title/Abstract])) OR (Traditional Tongue Diagnosis[Title/Abstract])) OR (Tongue Diagnoses, Traditional[Title/Abstract])) OR (Tongue Diagnosis, Traditional[Title/Abstract])) OR (Traditional Tongue Diagnoses[Title/Abstract])) OR (Traditional Tongue Assessment[Title/Abstract])) OR (Tongue Assessment, Traditional[Title/Abstract])) OR (Traditional Tongue Assessments[Title/Abstract])) OR (Medicine, chinese traditional[MeSH Terms])) OR (((((((((((((Herbal Medicine[Title/Abstract]) OR (Medicine, Herbal[Title/Abstract])) OR (Hawaiian Herbal Medicine[Title/Abstract])) OR (Hawaiian Herbal Medicines[Title/Abstract])) OR (Herbal Medicine, Hawaiian[Title/Abstract])) OR (Herbal Medicines, Hawaiian[Title/Abstract])) OR (Medicine, Hawaiian Herbal[Title/Abstract])) OR (Medicines, Hawaiian Herbal[Title/Abstract])) OR (La'au Lapa'au[Title/Abstract])) OR (Laau Lapaau[Title/Abstract])) OR (La au Lapa au[Title/Abstract])) OR (Herbalism[Title/Abstract])) OR (Herbal Medicine[MeSH Terms]))) OR ((((((((((((((((((Medicine, Traditional[Title/Abstract]) OR (Traditional Medicine[Title/Abstract])) OR (Home Remedies[Title/Abstract])) OR (Home Remedy[Title/Abstract])) OR (Remedies, Homeziy[Title/Abstract])) OR (Remedy, Home[Title/Abstract])) OR (Medicine, Primitive[Title/Abstract])) OR (Primitive Medicine[Title/Abstract])) OR (Medicine, Folk[Title/Abstract])) OR (Folk Medicine[Title/Abstract])) OR (Medicine, Indigenous[Title/Abstract])) OR (Indigenous Medicine[Title/Abstract])) OR (Folk Remedies[Title/Abstract])) OR (Folk Remedy[Title/Abstract])) OR (Remedies, Folk[Title/Abstract])) OR (Remedy, Folk[Title/Abstract])) OR (Ethnomedicine[Title/Abstract])) OR (Medicine, Traditional[MeSH Terms]))) OR ((((((((((((((((((((((((((((Acupuncture[Title/Abstract]) OR (Pharmacopuncture[Title/Abstract])) OR (Acupuncture Treatment[Title/Abstract])) OR (Acupuncture Treatments[Title/Abstract])) OR (Treatment, Acupuncture[Title/Abstract])) OR (Therapy, Acupuncture[Title/Abstract])) OR (Pharmacoacupuncture Treatment[Title/Abstract])) OR (Treatment, Pharmacoacupuncture[Title/Abstract])) OR (Pharmacoacupuncture Therapy[Title/Abstract])) OR (Therapy, Pharmacoacupuncture[Title/Abstract])) OR (Acupotomy[Title/Abstract])) OR (Acupotomies[Title/Abstract])) OR (Acupunctures, Ear[Title/Abstract])) OR (Ear Acupunctures[Title/Abstract])) OR (Auricular Acupuncture[Title/Abstract])) OR (Ear Acupuncture[Title/Abstract])) OR (Acupuncture, Auricular[Title/Abstract])) OR (Acupunctures, Auricular[Title/Abstract])) OR (Auricular Acupunctures[Title/Abstract])) OR (Acupuncture Point[Title/Abstract])) OR (Point, Acupuncture[Title/Abstract])) OR (Points, Acupuncture[Title/Abstract])) OR (Acupoints[Title/Abstract])) OR (Acupoint[Title/Abstract])) OR (Analgesia, Acupuncture[Title/Abstract])) OR (Acupuncture Anesthesia[Title/Abstract])) OR (Anesthesia, Acupuncture[Title/Abstract])) OR (Acupuncture[MeSH Terms]))) OR (((Moxibustion[Title/Abstract]) OR (Moxabustion[Title/Abstract])) OR (Moxibustion[MeSH Terms]))) OR ((((((((((Massage[Title/Abstract]) OR (Zone Therapy[Title/Abstract])) OR (Therapies, Zone[Title/Abstract])) OR (Zone Therapies[Title/Abstract])) OR (Therapy, Zone[Title/Abstract])) OR (Massage Therapy[Title/Abstract])) OR (Massage Therapies[Title/Abstract])) OR (Therapies, Massage[Title/Abstract])) OR (Therapy, Massage[Title/Abstract])) OR (Massage[MeSH Terms]))) OR (((((((Cupping Therapy[Title/Abstract]) OR (Cupping Therapies[Title/Abstract])) OR (Therapy, Cupping[Title/Abstract])) OR (Cupping Treatment[Title/Abstract])) OR (Cupping Treatments[Title/Abstract])) OR (Treatment, Cupping[Title/Abstract])) OR (Cupping Therapy[MeSH Terms]))) OR ((((Qigong[Title/Abstract]) OR (Qi Gong[Title/Abstract])) OR (Ch'i Kung[Title/Abstract])) OR (Qigong[MeSH Terms]))) OR ((((((((((((Tai Ji[Title/Abstract]) OR (Tai-ji[Title/Abstract])) OR (Tai Chi[Title/Abstract])) OR (Chi, Tai[Title/Abstract])) OR (Tai Ji Quan[Title/Abstract])) OR (Ji Quan, Tai[Title/Abstract])) OR (Quan, Tai Ji[Title/Abstract])) OR (Taiji[Title/Abstract])) OR (Taijiquan[Title/Abstract])) OR (T'ai Chi[Title/Abstract])) OR (Tai Chi Chuan[Title/Abstract])) OR (Tai Ji[MeSH Terms]))) OR ((((((((((((Therapies, Complementary[Title/Abstract])) OR (Therapy, Complementary[Title/Abstract])) OR (Complementary Medicine[Title/Abstract])) OR (Medicine, Complementary[Title/Abstract])) OR (Alternative Medicine[Title/Abstract])) OR (Medicine, Alternative[Title/Abstract])) OR (Alternative Therapies[Title/Abstract])) OR (Therapies, Alternative[Title/Abstract])) OR (Therapy, Alternative[Title/Abstract])) OR (Complementary Therapies[Title/Abstract])) OR (Complementary Therapies[MeSH Terms]))) AND ((((((((((((((((((((((((((((((Tension-Type Headache[Title/Abstract]) OR (Headache, Tension-Type[Title/Abstract])) OR (Headaches, Tension-Type[Title/Abstract])) OR (Tension Type Headache[Title/Abstract])) OR (Tension-Type Headaches[Title/Abstract])) OR (Idiopathic Headache[Title/Abstract])) OR (Headache, Idiopathic[Title/Abstract])) OR (Headaches, Idiopathic[Title/Abstract])) OR (Idiopathic Headaches[Title/Abstract])) OR (Stress Headache[Title/Abstract])) OR (Headache, Stress[Title/Abstract])) OR (Headaches, Stress[Title/Abstract])) OR (Stress Headaches[Title/Abstract])) OR (Tension Headache[Title/Abstract])) OR (Headache, Tension[Title/Abstract])) OR (Headaches, Tension[Title/Abstract])) OR (Tension Headaches[Title/Abstract])) OR (Psychogenic Headache[Title/Abstract])) OR (Headache, Psychogenic[Title/Abstract])) OR (Headaches, Psychogenic[Title/Abstract])) OR (Psychogenic Headaches[Title/Abstract])) OR (Tension-Vascular Headache[Title/Abstract])) OR (Headache, Tension-Vascular[Title/Abstract])) OR (Headaches, Tension-Vascular[Title/Abstract])) OR (Tension Vascular Headache[Title/Abstract])) OR (Tension-Vascular Headaches[Title/Abstract])) OR (New daily persistent headache[Title/Abstract])) OR (Contraction headache[Title/Abstract])) OR ("Tension-Type Headache"[Mesh]))

### Embase 2022/11/15

**#1** 'tension headache'/exp

**#2** 'headache, pressure':ti,ab OR 'headache, tension':ti,ab OR'pressure headache':ti,ab OR 'pressure headaches':ti,ab OR 'tension headaches':ti,ab OR 'tension type headache':ti,ab OR 'tension type headaches':ti,ab OR 'tension-type headache':ti,ab OR 'chronic tension headaches':ti,ab OR 'chronic tension type headache':ti,ab OR 'chronic tension type headaches':ti,ab OR 'episodic tension headaches':ti,ab OR 'episodic tension type headache':ti,ab OR 'episodic tension type headaches':ti,ab OR 'New daily persistent headache':ti,ab OR 'Contraction headache':ti,ab OR 'tension headache':ti,ab

**#3** 'Chinese medicine'/exp

**#4** 'Chinese herbal medicine':ti,ab OR 'medicine, Chinese traditional':ti,ab OR 'traditional Chinese medicine':ti,ab OR 'Chinese drug':ti,ab OR 'Chinese medicinal formulas':ti,ab OR 'fang ji fen lei':ti,ab OR 'fang-ji-fen-lei':ti,ab OR 'fangji fenlei':ti,ab OR 'fangji-fenlei':ti,ab OR 'fangjifenlei':ti,ab OR 'traditional Chinese medicinal formula':ti,ab OR 'traditional Chinese medicinal formulas':ti,ab OR 'Chinese medicine':ti,ab

**#5** 'traditional medicine'/exp

**#6** 'ethnomedicine':ti,ab OR 'folk medicine':ti,ab OR 'folk remedy':ti,ab OR 'indigenous medicine':ti,ab OR 'medicine, traditional':ti,ab OR 'native healing':ti,ab OR 'native medicine':ti,ab OR 'traditional healing':ti,ab OR 'traditional indigenous medicine':ti,ab OR 'traditional medicine':ti,ab

#7 'herbal medicine'/exp

**#8** 'botanical medicine':ti,ab OR 'herb medicine':ti,ab OR 'medicine, herbal':ti,ab OR 'medicine, herbal':ti,ab OR 'phyto-medicine':ti,ab OR 'phytomedicine':ti,ab OR 'plant medicine':ti,ab OR 'plant-based medicine':ti,ab OR 'herbal medicine':ti,ab

**#9** 'alternative medicine'/exp

**#10** 'alternative therapies':ti,ab OR 'alternative therapy':ti,ab OR 'complementary medicine':ti,ab OR 'complementary therapies':ti,ab OR 'diet fads':ti,ab OR 'eclecticism, historical':ti,ab OR 'mental healing':ti,ab OR 'mind body technique':ti,ab OR 'mind body therapies':ti,ab OR 'mind body therapy':ti,ab OR 'mind-body relations (metaphysics)':ti,ab OR 'mind-body relations, metaphysical':ti,ab OR 'mind-body therapies':ti,ab OR 'polarity therapy':ti,ab OR 'radiaesthesia':ti,ab OR 'radiesthesia':ti,ab OR 'reflexotherapy':ti,ab OR 'therapeutic cults':ti,ab OR 'alternative medicine':ti,ab

**#11** 'acupuncture'/exp

**#12** 'acupuncture therapy':ti,ab OR 'auriculotherapy':ti,ab OR 'point, acupuncture':ti,ab OR 'acupuncture, electric':ti,ab OR 'electric acupuncture':ti,ab OR 'acupuncture, ear':ti,ab OR 'acupuncture, earlobe':ti,ab OR 'ear acupuncture':ti,ab OR 'earlobe acupuncture':ti,ab OR 'acupuncture needle, single-use':ti,ab OR 'needle, acupuncture':ti,ab OR 'acupuncture anaesthesia':ti,ab OR 'acupuncture anesthesia':ti,ab OR 'analgesia, acupuncture':ti,ab OR 'acupuncture':ti,ab

**#13** 'moxibustion'/exp

**#14** 'Moxabustion':ti,ab OR 'moxibustion':ti,ab

**#15** 'massage'/exp

**#16** 'massage therapy':ti,ab OR 'masso-therapy':ti,ab OR 'massotherapy':ti,ab OR 'sports massage':ti,ab OR 'massage':ti,ab

**#17** 'cupping therapy'/exp

**#18** 'cupping (therapy)':ti,ab OR 'cupping manipulation':ti,ab OR 'cupping treatment':ti,ab OR 'fire cupping':ti,ab OR 'flash cupping':ti,ab OR 'moving cupping':ti,ab OR 'suction cupping':ti,ab OR 'vacuum cupping':ti,ab OR 'cupping therapy':ti,ab

**#19** 'qigong'/exp

**#20** 'chi kung':ti,ab OR 'chigung':ti,ab OR 'qigong':ti,ab

**#21** 'Tai Chi'/exp

**#22** 'Tai Chi Chuan':ti,ab OR 'Tai Ji':ti,ab OR 'Taiji quan':ti,ab OR 'Taijiquan':ti,ab OR 'Tai Chi':ti,ab

**#23** 'baduanjin':ti,ab OR 'tuina':ti,ab

**#24** #1 OR #2

**#25** #3 OR #4

**#26** #5 OR #6

**#27** #7 OR #8

**#28** #9 OR #10

**#29** #11 OR #12

**#30** #13 OR #14

**#31** #15 OR #16

**#32** #17 OR #18

**#33** #19 OR #20

**#34** #21 OR #22

**#35** #25 OR #26 OR #27 OR #28 OR #29 OR #30 OR #31 OR #32 OR #33 OR #34 OR #23

**#36** #24 AND #35

### 3 Cochrane library2022/11/15

**#1** MeSH descriptor: [Tension-Type Headache] explode all trees

**#2**  (Headache, Tension-Type):ti,ab OR (Headaches, Tension-Type):ti,ab OR (Tension Type Headache):ti,ab OR (Tension-Type Headaches):ti,ab OR (Idiopathic Headache):ti,ab OR (Headache, Idiopathic Headaches, Idiopathic):ti,ab OR (Idiopathic Headaches):ti,ab OR (Stress Headache):ti,ab OR (Headache, Stress):ti,ab OR (Headaches, Stress):ti,ab OR (Stress Headaches):ti,ab OR (Tension Headache):ti,ab OR (Headache, Tension):ti,ab OR (Headaches, Tension):ti,ab OR (Tension Headaches):ti,ab OR (Psychogenic Headache):ti,ab OR (Headache, Psychogenic):ti,ab OR (Headaches, Psychogenic):ti,ab OR (Psychogenic Headaches):ti,ab OR (Tension-Vascular Headache):ti,ab OR (Headache, Tension-Vascular):ti,ab OR (Headaches, Tension-Vascular):ti,ab OR (Tension Vascular Headache):ti,ab OR (Tension-Vascular Headaches):ti,ab OR (New daily persistent headache):ti,ab OR (Contraction headache):ti,ab(Word variations have been searched)

**#3** MeSH descriptor: [Medicine, chinese traditional] explode all trees

**#4** (Traditional Chinese Medicine):ti,ab OR (Chung I Hsueh):ti,ab OR (Hsueh, Chung I):ti,ab OR (Traditional Medicine, Chinese):ti,ab OR (Zhong Yi Xue):ti,ab OR (Chinese Traditional Medicine):ti,ab OR (Chinese Medicine, Traditional):ti,ab OR (Traditional Tongue Diagnosis):ti,ab OR (Tongue Diagnoses, Traditional):ti,ab OR (Tongue Diagnosis, Traditional):ti,ab OR (Traditional Tongue Diagnoses):ti,ab OR (Traditional Tongue Assessment):ti,ab OR (Tongue Assessment, Traditional):ti,ab OR (Traditional Tongue Assessments):ti,ab OR (medicine, Chinese traditional):ti,ab (Word variations have been searched)

**#5** MeSH descriptor: [Herbal Medicine] explode all trees

**#6** (Medicine, Herbal):ti,ab OR (Hawaiian Herbal Medicine):ti,ab OR (Hawaiian Herbal Medicines):ti,ab OR (Herbal Medicine, Hawaiian):ti,ab OR (Herbal Medicines, Hawaiian):ti,ab OR (Medicine, Hawaiian Herbal):ti,ab OR (Medicines, Hawaiian Herbal):ti,ab OR (La'au Lapa'au):ti,ab OR (Laau Lapaau):ti,ab OR (La au Lapa au):ti,ab OR (Herbalism):ti,ab (Word variations have been searched)

**#7** MeSH descriptor: [Medicine, Traditional] explode all trees

**#8** (Traditional Medicine):ti,ab OR (Home Remedies):ti,ab OR (Home Remedy):ti,ab OR (Remedies, Homeziy):ti,ab OR (Remedy, Home):ti,ab OR (Medicine, Primitive):ti,ab OR (Primitive Medicine):ti,ab OR (Medicine, Folk):ti,ab OR (Folk Medicine):ti,ab OR (Medicine, Indigenous):ti,ab OR (Indigenous Medicine):ti,ab OR (Folk Remedies):ti,ab OR (Folk Remedy):ti,ab OR (Remedies, Folk):ti,ab OR (Remedy, Folk):ti,ab OR (Ethnomedicine):ti,ab (Word variations have been searched)

**#9** MeSH descriptor: [Complementary Therapies] explode all trees

**#10** (Therapies, Complementary):ti,ab OR (Therapy, Complementary):ti,ab OR (Complementary Medicine):ti,ab OR (Medicine, Complementary):ti,ab OR (Alternative Medicine):ti,ab OR (Medicine, Alternative):ti,ab OR (Alternative Therapies):ti,ab OR (Therapies, Alternative):ti,ab OR(Therapy, Alternative):ti,ab (Word variations have been searched)

**#11** MeSH descriptor: [Acupuncture] explode all trees

**#12** (Pharmacopuncture):ti,ab OR (Acupuncture Treatment):ti,ab OR (Acupuncture Treatments):ti,ab OR (Treatment, Acupuncture):ti,ab OR (Therapy, Acupuncture):ti,ab OR (Pharmacoacupuncture Treatment):ti,ab OR (Treatment, Pharmacoacupuncture):ti,ab OR (Pharmacoacupuncture Therapy):ti,ab OR (Therapy, Pharmacoacupuncture):ti,ab OR (Acupotomy):ti,ab OR (Acupotomies):ti,ab OR (Acupunctures, Ear):ti,ab OR (Ear Acupunctures):ti,ab OR (Auricular Acupuncture):ti,ab OR (Ear Acupuncture):ti,ab OR (Acupuncture, Auricular):ti,ab OR (Acupunctures, Auricular):ti,ab OR (Auricular Acupunctures):ti,ab OR (Acupuncture Point):ti,ab OR (Point, Acupuncture):ti,ab OR (Points, Acupuncture):ti,ab OR (Acupoints):ti,ab OR (Acupoint):ti,ab OR (Analgesia, Acupuncture):ti,ab OR (Acupuncture Anesthesia):ti,ab OR (Anesthesia, Acupuncture):ti,ab (Word variations have been searched)

**#13** MeSH descriptor: [moxibustion] explode all trees

**#14** (Moxabustion):ti,ab(Word variations have been searched)

**#15** MeSH descriptor: [Massage] explode all trees

**#16** (Zone Therapy):ti,ab OR (Therapies, Zone):ti,ab OR (Zone Therapies):ti,ab OR (Therapy, Zone):ti,ab OR (Massage Therapy):ti,ab OR (Massage Therapies):ti,ab OR (Therapies, Massage):ti,ab OR (Therapy, Massage):ti,ab (Word variations have been searched)

**#17** MeSH descriptor: [Cupping Therapy] explode all trees

**#18** (Cupping Therapies):ti,ab OR (Therapy, Cupping):ti,ab OR (Cupping Treatment):ti,ab OR (Cupping Treatments):ti,ab OR (Treatment, Cupping):ti,ab OR (baguan):ti,ab (Word variations have been searched)

**#19** MeSH descriptor: [Qigong] explode all trees

**#20** (Qi Gong):ti,ab OR (Ch'i Kung):ti,ab (Word variations have been searched)

**#21** MeSH descriptor: [Tai Ji] explode all trees

**#22** (Tai-ji):ti,ab OR (Tai Chi):ti,ab OR (Chi, Tai):ti,ab OR (Tai Ji Quan):ti,ab OR (Ji Quan, Tai):ti,ab OR (Quan, Tai Ji):ti,ab OR (Taiji):ti,ab OR (Taijiquan):ti,ab OR (T'ai Chi):ti,ab OR (Tai Chi Chuan):ti,ab (Word variations have been searched)

**#23** (Baduanjin):ti,ab OR (tuina):ti,ab (Word variations have been searched)

**#24** (Tension-Type Headache):ti,ab (Word variations have been searched)

**#25** (Medicine, chinese traditional):ti,ab (Word variations have been searched)

**#26** (Herbal Medicine):ti,ab (Word variations have been searched)

**#27** (Medicine, Traditional):ti,ab (Word variations have been searched)

**#28** (Complementary Therapies):ti,ab (Word variations have been searched)

**#29** (Acupuncture):ti,ab (Word variations have been searched)

**#30** (moxibustion):ti,ab (Word variations have been searched)

**#31** (Massage):ti,ab (Word variations have been searched)

**#32** (Cupping Therapy):ti,ab (Word variations have been searched)

**#33** (Qigong):ti,ab (Word variations have been searched)

**#34** (Tai Ji):ti,ab (Word variations have been searched)

**#35** #1 OR #2OR #24

**#36** #3 OR #4 OR #25

**#37** #5 OR #6 OR #26

**#38** #7 OR #8 OR #27

**#39** #9 OR #10 OR #28

**#40** #11 OR #12 OR #29

**#41** #13 OR #14 OR #30

**#42** #15 OR #16 OR #31

**#43** #17 OR #18 OR #32

**#44** #19 OR #20 OR #33

**#45** #21 OR #22 OR #34

**#46** #36 OR #37 OR #38 OR #39 OR #40 OR #41 OR #42 OR #43 OR #44 OR #45 OR #23

**#47** #35 AND #46

### 4 Web of science2022/11/15

**#1** TS=(Tension-Type Headache OR Headache, Tension-Type OR Headaches, Tension-Type OR Tension Type Headache OR Tension-Type Headaches OR Idiopathic Headache OF Headache, Idiopathic Headaches, Idiopathic OR Idiopathic Headaches OR Stress Headache OR Headache, Stress OR Headaches, Stress OR Stress Headaches OR Tension Headache OR Headache, Tension OR Headaches, Tension OR Tension Headaches OR Psychogenic Headache OR Headache, Psychogenic OR Headaches, Psychogenic OR Psychogenic Headaches OR Tension-Vascular Headache OR Headache, Tension-Vascular OR Headaches, Tension-Vascular OR Tension Vascular Headache OR Tension-Vascular Headaches OR New daily persistent headache OR Contraction headache)

**#2** TS=(Medicine, chinese traditional OR Traditional Chinese Medicine OR Chung I Hsueh OR Hsueh, Chung I OR Traditional Medicine, Chinese OR Zhong Yi Xue OR Chinese Traditional Medicine OR Chinese Medicine, Traditional OR Traditional Tongue Diagnosis OR Tongue Diagnoses, Traditional OR Tongue Diagnosis, Traditional OR Traditional Tongue Diagnoses OR Traditional Tongue Assessment OR Tongue Assessment, Traditional OR Traditional Tongue Assessments)

**#3** TS=(Herbal Medicine OR Medicine, Herbal OR Hawaiian Herbal Medicine OR Hawaiian Herbal Medicines OR Herbal Medicine, Hawaiian OR Herbal Medicines, Hawaiian OR Medicine, Hawaiian Herbal OR Medicines, Hawaiian Herbal OR La'au Lapa'au OR Laau Lapaau OR La au Lapa au OR Herbalism)

**#4** TS=(Medicine, Traditional OR Traditional Medicine OR Home Remedies OR Home Remedy OR Remedies, Homeziy OR Remedy, Home OR Medicine, Primitive OR Primitive Medicine OR Medicine, Folk OR Folk Medicine OR Medicine, Indigenous OR Indigenous Medicine OR Folk Remedies OR Folk Remedy OR Remedies, Folk OR Remedy, Folk OR Ethnomedicine)

**#5** TS=(Complementary Therapies OR Therapies, Complementary OR Therapy, Complementary OR Complementary Medicine OR Medicine, Complementary OR Alternative Medicine OR Medicine, Alternative OR Alternative Therapies OR Therapies, Alternative OR Therapy, Alternative)

**#6** TS=(Acupuncture OR Pharmacopuncture OR Acupuncture Treatment OR Acupuncture Treatments OR Treatment, Acupuncture OR Therapy, Acupuncture OR Pharmacoacupuncture Treatment OR Treatment, Pharmacoacupuncture OR Pharmacoacupuncture Therapy OR Therapy, Pharmacoacupuncture OR Acupotomy OR Acupotomies OR Acupunctures, Ear OR Ear Acupunctures OR Auricular Acupuncture OR Ear Acupuncture OR Acupuncture, Auricular OR Acupunctures, Auricular OR Auricular Acupunctures OR Acupuncture Point OR Point, Acupuncture OR Points, Acupuncture OR Acupoints OR Acupoint OR Analgesia, Acupuncture OR Acupuncture Anesthesia OR Anesthesia, Acupuncture)

#7 TS=(moxibustion OR Moxabustion)

**#8** TS=(Massage OR Zone Therapy OR Therapies, Zone OR Zone Therapies OR Therapy, Zone OR Massage Therapy OR Massage Therapies OR Therapies, Massage OR Therapy, Massage)

**#9** TS=(Cupping Therapy OR Cupping Therapies OR Therapy, Cupping OR Cupping Treatment OR Cupping Treatments OR Treatment, Cupping OR baguan)

**#10** TS=(Qigong OR Qi Gong；Ch'i Kung OR Tai Ji OR Tai-ji OR Tai Chi OR Chi, Tai OR Tai Ji Quan OR Ji Quan, Tai OR Quan, Tai Ji OR Taiji OR Taijiquan OR T'ai Chi OR Tai Chi Chuan OR Baduanjin OR tuina)

**#11** #2 OR #3 OR #4 OR #5 OR #6 OR #7 OR #8 OR #9 OR #10

**#12** #1 AND #11 **(索引=SCI-EXPANDED, SSCI, A&HCI, CPCI-S, CPCI-SSH, BKCI-S, BKCI-SSH, ESCI, CCR-EXPANDED, IC 时间跨度=所有年份)**

1. **ClinicalTrials.gov**

**Condition or disease:** Tension-Type headache**。Status:** Recruitment, Suspended, Terminated, Completed, Unknown status; Expanded Access, Avaliable.
